# Sensors Fusion in Digital Healthcare Applications

**DOI:** 10.3390/s26061870

**Published:** 2026-03-16

**Authors:** Boon Giin Lee

**Affiliations:** School of Computer Science, University of Nottingham Ningbo China, Ningbo 315100, China; boon-giin.lee@nottingham.edu.cn

## 1. Introduction

Sensors constitute a fundamental element of modern digital healthcare, acting as the primary interface for capturing physiological, environmental, and biomechanical data from patients and clinical settings. The convergence of multiple sensing modalities, including wearable physiological monitors, environmental sensors, depth cameras, and inertial measurement units, has enabled a more comprehensive understanding of human health and disease processes [1,2,3]. This sensor fusion framework, coupled with advances in artificial intelligence, has opened new possibilities for personalized medicine, remote patient monitoring, and clinical decision support. However, the growing complexity and volume of healthcare data, together with the demands for real-time processing, privacy preservation, and model interpretability, present ongoing challenges that require innovative methodological approaches [4,5,6].

The intersection of sensor technologies and AI has accelerated the development of intelligent healthcare systems capable of automated detection, prediction, and intervention in various clinical domains. Sensor fusion approaches are transforming healthcare delivery and monitoring approaches, from assessing mental workload and detecting stress to analyzing performances in rehabilitation [7,8,9]. However, the main challenges remain, including ensuring data quality, addressing class imbalance, achieving hardware efficiency for wearable platforms, and maintaining the clinical interpretability of complex models [10,11,12]. The generation of high-quality synthetic data to augment limited real-world datasets, together with the benchmarking of wearable systems against gold-standard references, further highlights the multi-dimensional character of this research area.

This Special Issue, “Sensors Fusion in Digital Healthcare Applications”, brings together 11 original research articles and a comprehensive systematic review that address these challenges through novel sensor-integration strategies, advanced signal processing techniques, and clinically validated AI models. The collected contributions showcase the wide scope of sensor fusion applications, spanning the multimodal physiological monitoring in autism spectrum disorders, biomechanical assessments of stair descent, the synthetic generation of medical time series, and hardware acceleration for EEG processing. Together, they highlight the transformative promise of integrated sensing technologies for advancing digital healthcare throughout life.

## 2. Overview of the Published Papers

In the first contribution, the authors present a sensor- and machine learning-based sensory management recommendation system (SMRS) designed to assist children with autism spectrum disorder (ASD) in managing atypical sensory responses within classroom settings. The SMRS integrates multi-modal data from wearable and environmental sensors, including physiological monitors, accelerometers, and ambient light, noise, and temperature sensors, with machine learning models for the real-time detection of attention and stress levels. A novel aspect is the integration of a standardized sensory profile questionnaire to personalize the SMRS’s understanding of each child’s unique sensory processing patterns, alongside a fuzzy logic-based decision-making module that generates context-aware, real-time recommendations for caregivers and teachers. Through a comprehensive evaluation involving 30 children with ASD and 30 typically developing peers, the study demonstrates that SMRS can effectively identify distractions and stressors and that the implementation of its recommended strategies leads to statistically significant improvements in children’s attention and stress regulation. The results of a System Usability Scale questionnaire further indicate that the SMRS is perceived as user-friendly. The authors conclude that while the proposed SMRS represents a significant step toward low-cost, personalized, and real-time support for children with ASD.

In the second contribution, the authors explore the application of sensor fusion to optimize the trade-off between airborne pandemic control and energy consumption in digital indoor healthcare settings. The study uses a multi-sensor array consisting of air-quality monitors, temperature and humidity sensors, energy consumption meters, and 3D LiDAR scanners to characterize the physical and environmental dynamics of hospital inpatient rooms, classrooms, and conference rooms under different ventilation conditions. Through combining these heterogeneous sensor data, the authors construct high-fidelity input parameters for a computational fluid dynamics model coupled with an epidemiological simulation, allowing for detailed analysis of how open windows, pedestal fans, and air-conditioning systems influence airborne pathogen transmission. A Pareto front analysis using fused sensor datasets reveals that while all three ventilation solutions can be epidemiologically effective, certain configurations, such as low-speed air-conditioning, are highly energy-inefficient compared to high-speed fans. This work demonstrates how sensor fusion can inform evidence-based, multi-objective decision-making in digital public health infrastructure, balancing infection control with sustainability goals. Future research directions include the integration of real-time thermal comfort sensors to include human factors in the optimization framework and the development of room-level adaptive ventilation control systems driven by continuous sensor data streams.

In the third contribution, the authors present a sensor fusion-based methodology to estimate patient length of stay in intensive care units using data collected during the first 24 h after admission. The work integrates heterogeneous data from the MIMIC-III database, including monitors of vital signs at the bedside, laboratory test results, and demographic information, transforming raw variables into 139 statistical indicators. Using XGBoost as the core estimator, a novel two-step Bayesian optimization approach on GPU architecture automatically identifies the optimal hyperparameter configuration to minimize prediction error. Experimental results from 33,847 stays in the ICU demonstrate that the methodology achieves a mean absolute error of 2.529 days, significantly exceeding both the default XGBoost model (3.040 days) and the single-step optimization baseline (2.539 days), while also outperforming the current state-of-the-art generic LoS prediction models. The GPU-accelerated implementation reduces execution time by approximately 85% compared to multi-core CPU processing. The authors conclude that such automated machine learning systems driven by sensor fusion can serve as powerful digital healthcare tools for managing ICU occupancy and supporting clinical decision-making.

In the fourth contribution, the authors present a sensor fusion-based deep learning framework to assess stress levels in university students under ecologically valid problem-solving conditions. The study addresses a gap in stress detection research by designing an experimental protocol centered on Sudoku tasks in three distracting scenarios: exposure to discordant audio and horror videos, silent observation, and comforting stimuli. The study integrates multi-modal physiological data from wearable sensors, including PPG, ECG, and EEG, capturing responses associated with cognitive load and environmental stressors. The authors evaluated three advanced deep learning architectures, including StressNeXt, a long-term recurrent convolutional network (LRCN), and a self-supervised CNN, for stress classification. The findings demonstrate that sensor fusion, particularly the combination of ECG and EEG, enables the LRCN model to achieve high proficiency in discriminating stress levels, while StressNeXt shows exceptional performance under comforting conditions. The study further reveals that the ECG alone serves as a highly effective signal, whereas the EEG data are more susceptible to motion artifacts. The authors conclude that such sensor fusion-driven systems can provide valuable digital healthcare tools to monitor student well-being in academic settings.

In the fifth contribution, the authors present a sensor fusion-based data augmentation method to improve indoor localization in nursing care facilities using Bluetooth Low-Energy (BLE) beacons. The work addresses data imbalances in real-world healthcare settings, where certain areas are underrepresented due to variability in caregiving routines. Through combining RSSI data from 25 stationary beacons, the authors analyze signal patterns across semantic locations using standard deviation and KL divergence as similarity metrics. A novel relabeling approach identifies matching signal patterns between under-represented (minority) and well-represented (majority) room classes, enabling beacon data to be reassigned to augment minority class samples. Two variations are implemented: full matching, requiring complete surrounding beacon configurations, and partial matching for incomplete sensor coverage. The augmented data are used within a Random Forest classifier for room-level localization. The authors demonstrate that this relabeling strategy, particularly KL divergence with full matching, achieves better minority class classification and improves overall performance compared to conventional techniques. The study concludes that the use of existing sensor data through intelligent signal pattern analysis offers a practical and data-efficient approach to improving indoor positioning in nursing settings.

In the sixth contribution, the authors present a sensor fusion-based comparative study examining the impact of two cockpit display interface designs, which are the traditional Steam Gauge panel and the modern G1000 Glass panel, on novice pilots’ mental workload and situational awareness within a flight simulator environment. The system integrates multi-modal physiological sensing, combining EEG to capture the brain activity patterns associated with cognitive load, and HRV from electrocardiogram sensors to assess autonomic nervous system responses. Through integrating these objective physiological measures with subjective NASA-TLX assessments, the authors evaluated how different interface designs influence pilots’ cognitive states during simulated flight tasks with varying secondary task demands. The findings demonstrate that the G1000 Glass panel is more effective in supporting situational awareness and reducing mental workload compared to the distributed analog gauges, as evidenced by its significantly better flight performance and increased alpha band activity in frontal EEG channels. The study concludes that sensor fusion-driven cognitive state assessment provides valuable insight into human–computer interaction research in aviation, influencing the design of future cockpit interfaces.

In the seventh contribution, the authors present a systematic review of hardware acceleration techniques for CNNs applied to EEG signal analysis in digital healthcare applications, including emotion classification, motor imagery, epilepsy detection, and sleep monitoring. Unlike previous reviews that focused on software-based solutions, this work addresses the challenge of deploying CNN-based EEG analysis on resource-constrained wearable devices, where small form factor, low power consumption, real-time processing, and data security are critical. The review classifies the EEG feature extraction methods into five categories, including frequency-based, time-based, time–frequency, spatial features, and raw signals, and analyzes their suitability for hardware implementation on FPGAs, ASICs, and GPUs. The authors also examine efficient CNN design techniques adapted for EEG signals, including pruning, quantization, tensor decomposition, knowledge distillation, and neural architecture searches. Through synthesizing current research and identifying critical gaps, such as the need for standardized metrics, multimodal datasets, and embedded artifact preprocessing, this review offers a foundational roadmap for the development of hardware-accelerated EEG systems for real-world healthcare settings.

In the eighth contribution, the authors present QAMT, a novel LLM framework for generating quality-assured medical time-series data to address the challenges of limited data volume, poor quality, and privacy concerns in digital healthcare. The study integrates heterogeneous data from hospital sensors, including continuous vital signs and laboratory measurements, with static patient information. Through the construction of a health knowledge graph that combines domain expertise from open medical databases with instance-level information from MIMIC-III and eICU, QAMT uses a modular architecture combining a GAN-based module for generating continuous temporal data and an LLM-based retrieval-augmented generation module for discrete static events. A quality-assurance module uses the knowledge graph through chain-of-thought reasoning to enforce clinical constraints and validate variable dependencies. The authors demonstrate that this approach produces higher-fidelity synthetic data that outperform existing GAN- and LLM-based methods in fidelity, utility, and privacy metrics while preserving interpretability throughout the generation pipeline.

In the ninth contribution, the authors present a sensor fusion-based biomechanical study investigating the effects of lower-limb muscle fatigue on dynamic postural control during a descent down stairs. The study integrates kinetic data from four stair-embedded force plates with kinematic data from a ten-camera 3D motion capture system, allowing for the synchronized collection of ground reaction forces and joint angle trajectories. Through inducing multi-joint fatigue through a standardized sit-to-stand task with heel raises, the authors use one-dimensional statistical parametric mapping to analyze continuous waveform changes in joint kinematics and kinetics. The sensor fusion approach reveals that fatigue significantly impairs dynamic stability, as evidenced by the increased margin of stability and required coefficient of friction values in critical gait events. Compensatory adaptations include increased knee flexion angles, increased step width, reduced swing phase proportion, and enhanced ankle plantar flexion moments, suggesting neuromuscular redistribution in which the distal joints compensate for proximal muscle fatigue. The authors conclude that this integrated approach provides quantitative biomechanical evidence for the assessment of fall risk and can inform joint-specific interventions in rehabilitation and prevention programs.

In the tenth contribution, the authors present a sensor fusion-based approach to modernize the Fukuda Stepping Test for objective postural control assessment in older adults living in the community. The study uses a Microsoft Kinect v2 depth sensor to capture three-dimensional skeletal kinematics during stepping with the eyes closed, combining temporal, linear displacement and segmental angular parameters with established clinical measures of balance, mobility, cognitive function, physical activity and quality of life. Through replacing subjective visual estimation with automated kinematic tracking, the authors demonstrate that depth sensor-derived parameters, particularly trunk flexion and upper trunk rotation, exhibit clinically meaningful associations with cognitive performance and functional mobility that substantially exceed traditional displacement-only metrics. The sensor fusion approach reveals that excessive trunk flexion may reflect impaired cognitive–motor integration, while increased head mediolateral displacement correlates with frailty and fear of falling. The authors conclude that integrating markerless motion capture technology into geriatric assessment protocols provides objective, multidimensional information on postural control mechanisms, enabling more precise fall-risk screening and individualized rehabilitation targeting in aging populations.

In the eleventh contribution, the authors present a validation study of an IMU-based method to estimate the kinematics of three-dimensional lower limb in asymptomatic individuals and patients with gait disorders, including cerebral palsy. The sensor fusion approach integrates data from seven IMUs positioned on the pelvis, thighs, shanks, and feet, synchronizing accelerometer and gyroscope signals with a reference optoelectronic motion capture system. Through combining inertial data through advanced orientation estimation and sensor-to-segment alignment algorithms, the authors demonstrate that IMU-derived kinematics achieve good to excellent correlation with the reference measures, with mean root mean square errors below 10° and centered errors below 3.2° after offset removal. Reliability analysis reveals moderate to excellent intra- and inter-operator agreement for sagittal- and frontal plane kinematics. A comparison of the gait profile scores of both methods shows moderate agreement, with no statistically significant differences in pathological populations. The authors conclude that this methodology provides a clinically viable and accessible alternative to traditional gait laboratories to characterize impairments and support therapeutic decision-making.

## 3. Conclusions

This Special Issue presents 10 original research articles and one comprehensive review that collectively showcase the transformative potential of sensor fusion in digital healthcare. Contributions span various domains, including neurodevelopmental disorders, assessment of mental workload, indoor localization, biomechanical analysis, and synthetic data generation, demonstrating how multimodal sensor integration combined with advanced AI enables personalized monitoring, clinical decision support, and improved patient outcomes. Several recurring themes appear throughout the collection: the significance of context-aware systems that adapt to individual characteristics, the importance of addressing data quality and imbalance through innovative methodologies, and the central role of hardware efficiency and interpretability in enabling practical, real-world deployment.

We hope these selected papers offer valuable insight into the methodological innovations and practical applications shaping the future of sensor-based digital healthcare, serving as both a reference for current best practices and an inspiration for future research in this rapidly evolving field.

We extend our sincere gratitude to all authors who contributed their outstanding research to this Special Issue, as well as to the dedicated reviewers whose rigorous and constructive feedback ensured the high quality of the published works. Their collective efforts have made this comprehensive overview of sensor fusion in digital healthcare possible.
